# The experience of employees using telework as an accommodation for health-related reasons: the importance to focus on the employee–manager relationship

**DOI:** 10.3389/fpubh.2026.1786980

**Published:** 2026-06-22

**Authors:** Alexandra Lecours, Lauriane Drolet, Alex-Anne Beaulieu, Roxanne Bédard-Mercier, Joanie Maclure

**Affiliations:** 1 Department of Occupational Therapy, Université du Québec à Trois-Rivières, Trois-Rivières, QC, Canada; 2 Center for Interdisciplinary Research in Rehabilitation and Social Integration, Québec, QC, Canada

**Keywords:** caregivers, chronic illness, employee–manager relationship, leader-member exchange (lmx) theory, telework, workforce, workplace accommodation

## Abstract

**Background:**

Workers living with chronic illness or fulfilling caregiving responsibilities may encounter specific challenges in maintaining employment, often necessitating workplace accommodations. The rise of telework following the COVID-19 pandemic has drawn attention to its potential as an accommodation measure, notably by offering greater flexibility, facilitating work–life balance, and reducing physical and logistical barriers to job retention.

**Methods:**

This qualitative study examined the experiences of individuals with chronic illness or caregiving responsibilities who use telework as an accommodation, with specific attention to the employee–manager relationship within this context. Using an interpretive descriptive design, two focus group discussions were conducted with 12 participants. Among participants, eight identified as workers with chronic illness, and four as caregivers.

**Results:**

Four key themes emerged regarding challenges in the employee–manager relationship: negotiation of accommodations, management of telework, clarification and respect of boundaries, and maintenance of social connections. Findings underscore the importance of the quality of the employee–manager relationship and the critical role of trust, as framed within leader-member exchange (LMX) theory.

**Conclusions:**

Strengthening trust and addressing workplace stigmas related to health status are crucial to fostering equitable telework accommodations, particularly in the context of labor shortages. Raising organizational awareness to break stereotypes and promote inclusivity is essential. Further research is needed to assess telework as a flexible and inclusive solution to support workers with health-related challenges or caregiving responsibilities.

## 1 Introduction

Many workers manage significant daily limitations related to chronic diseases. In Canada, nearly 44% of adults aged 20 and older report suffering from at least one common chronic condition, such as arthritis, diabetes, or cancer (1). Although these conditions are not always formally disclosed as disabilities, they impose real constraints, particularly through persistent pain and fatigue (2, 3). Work limitations can also stem from a worker's social responsibilities. In Quebec, one-third of adults act as caregivers, and 57% of them balance this role with employment (4). This dual reality increases the risk of stress and isolation, creating a role conflict that undermines work-life balance and compromises professional engagement (5–8). For these groups of workers, telework appears to be a valuable and adaptable solution for promoting sustained workforce participation and long-term job retention.

Since the COVID-19 pandemic, telework has become a new normal way of working for many employees. The use of various communication and information technologies has allowed employees to work outside the usual office space, mainly from home (9). An analysis carried out by Statistics Canada (10) indicates that nearly 25% of Canadians were still working exclusively from home or in a hybrid mode as of November 2023, compared to only 4% in 2016 (11). Although it is now widespread, telework has been used for nearly 30 years as an exceptional accommodation for people with physical disabilities (12). However, employers were often reluctant to resort to this measure as an accommodation at a larger scale (13). A literature review showed that managers may have concerns about the cost of telework implementation and that they sometimes lack trust in employees to adopt this mean of work delivery (14).

Several benefits of telework have been highlighted in recent years. Commonly cited advantages of telework are schedule flexibility (15), commute times savings (16) and increased job satisfaction (17). A literature review on the characteristics of telework before and after the pandemic concludes that it has enhanced employees' social and personal lives by promoting a healthier work-life balance (18). Overall, employees reported a positive experience and expressed a desire to continue this work mode on a permanent basis (19). A flexible arrangement of 2 or 3 days of telework per week seems to be the desirable adjustment to counterbalance the negative effects of telework, such as challenges in communication and knowledge sharing (20).

Telework is not a panacea, and some authors urge caution (21, 22). Telework can reduce a person's effectiveness in managing both family and professional activities due to the overlap between professional and private spheres (21). Similarly, lack of boundaries can lead to intensified work, including difficulties in disconnecting, leading to social isolation (23) and decrease in wellbeing (22, 24, 25). In another study, it was shown that teleworkers experience greater work-related fatigue compared to those who work on-site, possibly due to an increase in role conflicts (26). Among the other disadvantages of telework, impacts on ergonomics as well as on stress, anxiety, and loneliness levels in the general population have been reported (18). It should be noted that while limited research has focused on the negative effects of telework specifically for individuals with health-related issues, some evidence suggests that workers with mental health disorders may face a greater risk of stigma when using telework as an accommodation because of their health situation, particularly if the rest of the team is working on-site (27).

Despite these challenges, telework may be an option to enhance accommodation and inclusion for individuals with limitations (28–31). To our knowledge, few studies have explored this option in the post-pandemic era. Moreover, most recent publications focus on telework for all employees, rather than specifically as an accommodation for individuals with health-related issues (32). Some exploratory work, although, suggest positive outcomes. For example, a recent study conducted with autistic people suggest that they appreciate the reduction of interpersonal interactions and sensory stimulation that telework provide (33). For people with physical disabilities, schedule flexibility offered by telework allows for better control over their environment and working hours, which contributes to improved work performance, health, and management of their disability (34).

Various factors appear to influence access to telework as an accommodation measure. From a legal perspective, an employer has an obligation to provide accommodation by adjusting the working conditions and workplace to meet the specific needs of an individual with a permanent or temporary disability. This disability may pertain to physical, psychological, or social aspects of health (35). Managers also hold a moral responsibility to ensure employees' wellbeing (36). However, despite its accommodating nature, telework is not an inherent right. Even in cases involving significant limitations or specific personal circumstances, employers may still require on-site presence when job responsibilities demand it. In Quebec, section 20 of the Charter of Human Rights and Freedoms provides for an exception to the prohibition on employment discrimination if providing a reasonable accommodation would impose an undue burden. For example, a recent court decision upheld the denial of full-time telework for a guidance counselor with a physical disability causing significant mobility limitations, finding that in-person presence was essential to her role and that permanent telework would create undue hardship given the position's on-site requirements (37).

Beyond legal and practical considerations, access to and success of workplace accommodation is strongly shaped by managerial factors, particularly the quality of the employee–manager relationship. Accommodation decisions are often influenced by managers' subjective evaluations of employees, where perceived merit and likability can play a decisive role. In this context, managers' personal values and beliefs also shape how accommodations are interpreted and implemented (38). Leadership qualities such as empathy and openness to inclusion further facilitate access, as they foster a safe environment in which employees feel comfortable disclosing their needs without fear of reprisal (39–41). Overall, a strong, trust-based relationship between employees and managers emerges as a determinant of whether accommodation is both granted and effectively implemented.

## 2 Literature review

### 2.1 Workers with chronic illness

Individuals with chronic illness, also known as non-communicable diseases, have one or more conditions that are generally long-lasting and have developed due to a combination of genetic, physiological, environmental, and behavioral factors. These include mental health disorders, cardiovascular diseases, migraines, diabetes, or cancers (42). In 2021, nearly half of Canadians (45.1%) were living with at least one major chronic illness (43). Since chronic illness forms a heterogeneous group, symptoms are often variable and may even be invisible (44), which can affect work performance (3, 45).

Due to their lasting and evolving nature, chronic illness can lead to significant personal and professional challenges. For instance, individuals with chronic illnesses are at greater risk of experiencing mental health problems such as anxiety, depression, burnout, and psychological trauma (46, 47). This could be attributed, in part, to the significant responsibilities they must undertake to manage their condition. Five major responsibilities associated with being a worker and having a chronic illness have been highlighted, including: (1) self-managing physical and psychological symptoms; (2) navigating relationships with employers, family, friends, and healthcare providers; (3) handling finances, insurance, and medication needs; (4) scheduling and managing healthcare appointments; and (5) gathering information and conducting research regarding their illness (48).

Chronic illness can also limit career choices, reduce promotion opportunities, and lead to lower wages (49). Moreover, individuals with chronic illness are more likely to retire early from the workforce (50). A study found that each additional chronic illness reduces working life by one year when present at age 50, and by 0.7 years when present at age 60 (50). Workers having chronic illness often face various work limitations, including reduced productivity attributed to higher rates of presenteeism and absenteeism (51, 52), a slower work pace (53), and difficulties in completing tasks (3). Evidence shows that these workers may require more frequent breaks (54). Notably, a study conducted among employees with arthritis and/or diabetes revealed that it is common for these individuals to work while experiencing fatigue, pain, and fluctuating health conditions (55). Other obstacles for workers with chronic illness include commuting challenges and difficulties in communication with managers and colleagues (3). In fact, a study on workplace barriers faced by individuals having chronic conditions found that they frequently encounter misconceptions about their illness; participants reported that colleagues and managers sometimes question their professional abilities due to their condition, potentially hindering career advancement (56). Despite these challenges, individuals having chronic illness value work as it allows them to contribute to society, achieve financial independence, and maintain social connections (57).

Fortunately, technological advancements have made telework increasingly accessible, positioning it as a valuable accommodation option for individuals having chronic illness. Telework can help reduce barriers related to pain and fatigue associated with in-person work (12, 58). Additionally, teleworking may contribute to lower absenteeism rates. A survey of 98 workers with chronic illness reported that 77% agreed that working from home allowed them to miss less work due to the impacts of their medical condition (59). Given the association between unmet accommodation needs and work limitations among workers having chronic illness (55), the use of telework may alleviate challenges (e.g., concentration issues, reduced work pace, commuting difficulties) and offer substantial benefits by enabling greater control over work environment, hours, and breaks (57). Furthermore, telework can also benefit organizations by helping to delay retirement age, allowing them to retain experienced employees longer (60, 61).

### 2.2 Caregiving workers

Another group of workers that could benefit from telework are caregivers (31). Caregivers are individuals who provide support to one or more loved ones facing physical, psychosocial, or other disability, whether permanent or temporary, regardless of the care recipient's age, living situation, or the relationship between them (62). In the province of Quebec (Canada), one-third of adults take on caregiving responsibilities. Of these, 46% are employed full-time, while 11% work part-time (4). The support that caregivers provide takes various forms, such as giving care or accompanying loved ones to medical appointments. Nearly 31% of individuals aged 45 to 64 act as caregivers, while this proportion is around 21% among those aged 65 and older (63).

The role of a caregiver often comes with significant challenges. Studies revealed that caregivers are more likely to experience high levels of stress and feelings of isolation (5, 8) and they might struggle with establishing work-life balance between their professional responsibilities and caregiving duties (6). Increasing caregiving responsibilities have been associated with reduced time and energy for work, exacerbating perceptions of role conflict and leading to significant strain (7). The resulting physical and emotional strain can negatively impact caregivers' professional duties, particularly by reducing their productivity (64, 65). For instance, these individuals often have a high rate of absenteeism, including being late or leaving work early (64, 65) and report more work-related impairments (66).

Nonetheless, being employed while serving as a caregiver appears to have positive effects, providing a form of respite that enables individuals to recharge mentally (67). It seems that work is an important variable that can have positive effects on caregivers if it is accompanied by accommodation measures. Offering flexible work arrangements is the most frequent approach to supporting employed caregivers (68). This includes options such as flexible schedules, compressed workweeks, part-time work, reduced hours, and teleworking (69, 70). Employees greatly value flexibility from their employers as it helps them navigate the uncertainty inherent in caregiving situations (71). A study conducted with caregivers of individuals with cancer shows that those who have flexible schedules experience lower levels of stress compared to those without such flexibility (72).

Much like individuals with chronic illness, caregivers could benefit from telework as it allows them to provide care while staying connected to their workplace, actively participating in meetings, and accessing online training (73). However, few studies have explored the use of telework as an accommodation for caregivers. A study in Peru found a strong significant (*p* < 0.05) and positive relationship (ρ = 0.835) between telework and job placement, suggesting that telework significantly promoted the employment of caregivers of people with severe disabilities (74).

### 2.3 The manager's role in implementing accommodation measures

Current evidence indicates that the relationship between employees and managers significantly affects access to the benefits of accommodations, such as telework (75). For instance, effective communication and openness from managers are generally associated with higher success rates of implemented accommodations (76, 77). While positive relationships and effective communication with managers can facilitate successful accommodation, employees with health-related issues often face additional challenges due to biases or negative perceptions from managers (75). For example, managers' expectations regarding the performance of individuals with disabilities are typically lower (78). Such perceptions can negatively impact the quality of the employee–manager relationship (79) and affect these employees' work experience (75). The quality of the relationship between employees with autism and their managers has been associated with their success within the organization (80). A 2020 study revealed that increased severity of disability is associated with more negative work experiences for employees (75). The findings also indicate that while the severity of disability does not have a direct impact on the provision of job accommodations, the quality of the supervisor-employee relationship is positively correlated with the likelihood of an accommodation being provided (75). The decision to grant an accommodation may depend more on the manager's “willingness” to provide it rather than its “necessity.”

Similar findings have been observed among caregiver employees, with access to work-family balance measures depending almost exclusively on the relationship with the manager (81). A qualitative study conducted with caregiver workers highlights that obtaining an accommodation requires considerable effort and negotiation (70). The authors specified that the accommodation measures tend to be temporary, precarious, and heavily reliant on the discretion of the immediate supervisor, the support of colleagues, the terms of the collective agreement, or the stance of human resources. In fact, despite the benefits of flexible accommodations, such as improved morale, increased loyalty and stronger commitment to the organization, employers are often reluctant to offer telework (82). Certain employees' characteristics can also influence access to accommodations. A study found that those with greater seniority and less specialized roles are more likely to have their accommodation requests approved (83).

However, in the context of labor shortages, employer support and flexibility appear to be particularly important to ensure the sustained participation of skilled workers (84). To achieve this, it is essential to identify strategies that promote the inclusion and active engagement of all qualified individuals, including individuals having chronic illness and caregivers. Yet, there is a significant gap in research addressing the specific challenges faced by these groups, particularly in relation to telework as a feasible accommodation.

## 3 Theoretical framework

### 3.1 Leader–member exchange (LMX) theory

Leader-member exchange (LMX) theory asserts that each dyadic relationship between a manager and an employee is distinct, and that this relationship significantly impacts the leadership style the manager applies to the employee (80, 85), notably regarding accommodation and telework. These employee–manager relationships develop through unique social exchanges between a manager and each of their employees (80, 86). Leadership making has been conceptualized as a three-stage process: (1) the stranger phase, (2) the acquaintance phase, and (3) the mature partnership phase (87). The first phase corresponds to the initial context where the manager assesses the employee's skills and potential contributions (88). This allows the manager to determine whether they assign new roles and responsibilities to the employee (88–90). At this stage, their interactions are contractual and strictly work-related. The second phase represents the period during which the manager and the employee begin to understand one another and possibly develop mutual trust (90). At this stage, interactions are still heavily anchored in the employment contract, but occasional personal exchanges emerge, and reciprocal favors are observed. This phase is considered a testing period in which the relationship between the two parties may develop positively or negatively (88, 89). When the relationship develops negatively, various consequences can be identified in the dynamic. In such situations, the manager tends to rely more on formal organizational authority (90), and employees are more likely to be given less engaging tasks (91). This phase is also marked by reduced interactions with the manager (91), increased negative exchanges when they do occur (92), heightened stress, role conflicts, age-based discrimination, decreased autonomy at work, and underutilization of the employee's skills (93). When the relationship develops positively, it progresses to a third phase known as the mature partnership. This partnership is distinguished by high-quality leader-member exchanges and characterized by loyalty, mutual support, respect, and trust (89). Employees who have this type of relationship with their manager receive more attention, information, and resources, which may include accommodations, compared to those in lower-quality exchanges. These employees are also better positioned to negotiate their work tasks, benefit from greater flexibility, and accommodate their family needs with minimal adjustments (94). Other positive effects of a high-quality relationship include higher employee engagement, greater satisfaction with management, and improved competence (95).

### 3.2 Employee–manager relationship quality in telework

Several studies explored the quality of employee–manager relationships in the context of telework using LMX theory, but the findings are mixed. For example, one study found that physical distance is negatively related to the quality of the relationship (96), while other studies have suggested that telework is not a barrier to developing high-quality relationships as long as electronic communication tools are effectively utilized (97, 98). While accessibility to the manager through technological tools is important, it is not the primary factor determining relationship quality. Researchers suggested that the critical element is how these tools are utilized. For example, managers who embody authentic leadership by self-disclosing and sharing knowledge through electronic communication are more likely to build high-quality exchanges with their employees and foster a healthy workplace climate (99).

## 4 Purpose of the study

In the current landscape of labor shortages and with increasing societal commitment to accommodating diverse worker needs, it is essential to identify strategies that promote the active participation of all qualified individuals. Access to work is especially important for populations facing health challenges, including individuals having chronic illness and caregivers, who may benefit significantly from telework as an accommodation. However, limited research exists on the experiences of these groups, particularly concerning the role of telework as a practical accommodation. Given the potential advantages of telework for individuals managing health-related issues or caregiving responsibilities and the influence of the employee–manager relationship in facilitating these arrangements, this study seeks to examine the experiences of individuals with chronic illness or caregiving responsibilities who utilize telework as an accommodation, with specific attention to the employee–manager relationship within this context.

## 5 Methods

### 5.1 Design

This study employed an interpretive descriptive design (100, 101), chosen for its capacity to explore a phenomenon from the perspective of individuals, identify its key components, and elucidate the relationships between these components. This approach is particularly suited to generate a deep understanding of a phenomenon within its natural context (102–104), making it appropriate for the aims of the present study.

### 5.2 Participants and procedure

Considering that employees have a unique perspective on the quality of their relationship with their manager (95, 105) and that little research exists on how employees perceive the accommodations they receive (106), this study sought to amplify employee voices by capturing their lived experiences. Participants were eligible for inclusion if they were at least 18 years old, capable of participating in a Zoom meeting, and met the following criteria: (1) employed for at least 5 years, (2) engaged in telework for at least 2 years, and (3) fit one of the study's focus categories, either (a) living with a chronic illness or (b) serving as a caregiver. A non-probability convenience sampling strategy (107) was employed to reach the target population via LinkedIn and Facebook. This strategy served as a timely and effective tool for acquiring nuanced insights while ensuring that the complex health and logistical realities of the participants were fully captured (108, 109). In line with methodological recommendations, the study aimed to recruit between nine (110) and twelve participants (111). This number aligns with established qualitative precedents in focus group research concerning the work participation of employees with chronic conditions (112, 113).

Data collection was conducted between May and June 2023. Prior to participation, participants were required to complete a questionnaire to collect sociodemographic data (e.g., age, gender, years of work experience, etc.). Following this, two focus groups were conducted on Zoom. The choice of a virtual format allowed for the inclusion of participants with chronic health conditions and caregiving duties by overcoming logistical barriers and providing an accessible and flexible environment (114). Focus groups were facilitated by a guide designed specifically for this study. It was also pretested with two workers who met the study's inclusion criteria. It aimed to explore participants' lived experiences, covering topics such as work practices (e.g., “In your situation, what work practices are used to promote your accommodation, inclusion, and health?”), the use of accommodations (e.g., “Tell me about the accommodations you have in place for your remote work. What challenges do these accommodations present?”), the relation with their manager (e.g., “How does your manager support your professional development and career growth?”) and the impact of telework on balancing roles (e.g., “Tell me about how working from home has affected the balance between your different roles in life.”). Several questions were posed to the group. The focus group discussions were audio-recorded and had an average duration of 111 min.

A total of twelve participants took part in one of the two focus groups. This final sample size was determined based on the principle of thematic saturation (115). Following the analysis of the second focus group, the coding reached a state of informational redundancy, as no novel themes or categories were identified beyond those established in the first group (116). Participants shared a comparable socio-professional context and were therefore considered a single demographic stratum, as defined by Hennink et al. (116). The sample homogeneity facilitated rapid thematic convergence, supporting the decision to use two focus groups to capture the nuances of the participants' experiences (117). These results are consistent with empirical evidence showing that the vast majority of themes are identifiable within the first two to three focus groups (118).

### 5.3 Data analysis

The audio recordings were first transcribed. The analysis proceeded in two sequential phases combining an inductive approach and a subsequent theoretical interpretation. The researchers first conducted an inductive thematic analysis to ensure that findings remained grounded in participants' experiences. They then mobilized LMX theory as an interpretive lens to explain the relational dynamics identified.

The inductive phase followed a bottom-up strategy in which themes emerged directly from the data without predefined categories (119, 120). The analysis followed a five-step strategy (121): (1) repeated reading of the transcripts to allow the researchers to immerse themselves in the data; (2) assignment of descriptive codes to meaningful units to develop an initial coding structure; (3) transformation of these units into expressions representative of the participants' lived experiences; (4) synthesis of these expressions to organize the data into a general structure, grouping codes into categories and emergent themes; and (5) iterative comparisons between the raw data and the analyzed data to refine the emerging structure

QDA Miner Lite supported data management. Two researchers independently coded each focus group under the supervision of the lead researcher. They compared and consolidated their analyses after each coding round to produce a shared version. This process strengthened interrater consistency (122). The lead researcher reviewed all coding decisions at each stage. The team repeatedly revisited the data, which enhanced analytic rigor and ensured a faithful representation of participants' accounts.

The second phase introduced LMX theory to interpret emergent themes (123). This step did not constrain the initial analysis. It provided a structured framework to explain the relational mechanisms observed within the employee–manager dyad. This integration clarified how telework as an accommodation operates in contexts involving chronic illness or caregiving responsibilities.

## 6 Results

### 6.1 Participants description

The sample consisted of 12 participants, of whom 10 were women and 10 held a university degree. The predominance of women in this sample aligns with Canadian data showing they disproportionately assume caregiving roles and report significantly higher accommodation needs (124, 125). Participants' ages ranged from 25 to 63 years. Regarding their status, eight identified as workers with chronic illnesses (e.g., diabetes, Crohn's disease, arthritis), while four were caregivers (e.g., for a parent with an illness or a disabled spouse). Their professional backgrounds were distributed across the public (*n* = 5), community (*n* = 4), and private (*n* = 3) sectors. Table 1 summarizes the sociodemographic and professional characteristics of the participants.

**Table 1:** Demographic and professional profile of participants.

ID	Gender	Age	Situation	Education level	Profession	Employment sector	Telework (%)	Union status	Organization size	Formal telework policy
P01	Female	33	Chronic illness	University	Project coordinator	Community	98	Non-unionized	< 50	No
P02	Female	59	Chronic illness	University	Translator	Private	50	Non-unionized	< 50	No
P03	Female	38	Chronic illness	University	Community information specialist	Community	100	Non-unionized	< 50	No
P04	Female	35	Chronic illness	University	Talent acquisition specialist	Private	100	Non-unionized	>1,000	Yes
P05	Female	31	Chronic illness	University	Virtual assistant	Private	100	Non-unionized	< 50	Yes
P06	Female	51	Chronic illness	University	Project manager	Community	95	Non-unionized	< 50	No
P07	Female	63	Chronic illness	University	Manager	Community	80	Non-unionized	< 50	No
P08	Female	28	Chronic illness	University	Senior HR assistant	Public	90	Non-unionized	>1,000	Yes
P09	Male	59	Caregiving	University	IT Consultant	Public	100	Non-unionized	Between 251 et 1,000	Yes
P10	Female	25	Caregiving	College	Social work technician	Public	50	Unionized	>1,000	Yes
P11	Female	55	Caregiving	College	Administrative technician	Public	60	Unionized	>1,000	Yes
P12	Male	56	Caregiving	University	Research officer	Public	40	Unionized	>1,000	Yes

### 6.2 Challenges faced by teleworkers in the employee–manager relationship

The study identified four major themes of challenges faced by employees using telework as an accommodation for health-related issues regarding the employee–manager relationship, which can be further divided into six specific categories. These results are presented in Table 2.

**Table 2:** Challenges faced by teleworkers in the employee–manager relationship.

Themes	Categories
1. Negotiation of accommodations	1.1 Negotiation between employees and their manager
	1.2 Manager's negotiation with the work team
2 Management of telework	2.1 Expectation of compassionate oversight by the manager
3 Clarification and respect of boundaries	3.1 Differentiation between telework, sick leave, and vacation
4 Social connections	4.1 Trust in relationships
	4.2 Respect of privacy

#### 6.2.1 Negotiation of accommodations

The results show that the implementation of telework as an accommodation often requires negotiation between the different parties involved. This negotiation first takes place between workers and their manager regarding the realistic teleworking arrangements for each party (Category 1.1.). Participants highlighted that they had to put pressure on their manager or assert their need for accommodation to get approval. This pressure was often applied to prompt quicker decisions from managers regarding accommodation requests. Indeed, when workers see an opportunity to enhance their health, they are likely to persistently advocate for these accommodations with their manager:

I still had to push a lot, constantly following up with my manager, even though I was on leave, to get answers and ensure there was a follow-up to this. It took several months, but it got done. [...] Then it greatly helped my rehabilitation, but it wasn't without effort on my part. I think that, in fact, if I hadn't insisted, I wouldn't have received that equipment. (P04)^1^

^1^

Similarly, for some workers, securing an accommodation can be a pivotal moment in aligning their employment with their health needs. Therefore, even if the requested accommodation is not a standard practice within the organization, some will still attempt to negotiate it with their manager:

It was a small company and at one point, I had to negotiate my contract for 100% telework, but that part was a bit difficult for them. [...] So, I think I twisted the boss's arm a bit there [...], and for me, it was clear that if I didn't get that, well, I wouldn't accept the contract. (P09)

This negotiation between the involved parties generally requires adaptability from both the worker and the manager, as it often involves deviating from what is typically common within the company. The participants' comments show different attempts to adapt to the contexts that arise in the organization of telework accommodations. These adjustments often manifest in how work tasks are organized. For example, a manager might support a worker's focus on a particular task by temporarily relieving them of their regular responsibilities: “Often [the manager] adapts by giving, for example, a day off where we do more writing to actually progress on our reports” (P10).

At other times, the worker and manager adjust their work schedules to prioritize an important meeting between them: “And if there are urgent matters, we will always move it to the next day or find another time; we won't just cancel a meeting and not talk at all” (P04).

Finally, participants' statements show that the agreement on accommodation fosters the worker's sense of belonging to the organization. Indeed, a positive response from the manager to the worker's request can translate into recognition and involvement in the organization on the part of the worker:

So, I negotiated with my employer to start [work] later, and she welcomed my request with open arms. That really made me, like, I am very grateful to this day, and I stay with this company precisely because of that, for their humanity, because my employer even took the trouble to hire another person to cover the hours I didn't work in the morning. And so, I have no issues in that regard; I really, I love my job, my employer, and I am very happy despite my illness. (P05)

Secondly, the results reveal that managers often engage in a level of negotiation with the work team when implementing telework as an accommodation for employees with health concerns (Category 1.2.). Such accommodation can sometimes lead to tensions within the team, as colleagues may view them as instances of favoritism or special treatment: “[My accommodation situation] is starting to create tensions within the team because what I do and what others do is not the same” (P06).

#### 6.2.2 Management of telework

Participants' discussions show that workers expect compassionate and supportive oversight from managers (Category 2.1.). For workers with chronic illness or caregiving responsibilities, experiencing genuine interest and concern from their manager contributes significantly to their overall wellbeing:

I believe that the key element is humanity, putting oneself in the shoes of the employees […] that's what makes me feel very comfortable, even very happy. It doesn't take much to understand that another person is suffering from something and needs a certain accommodation. So, I think that's the key, it's humanity overall. (P05)

This genuine interest can be expressed in numerous ways. Participants mentioned, for instance, simply checking in with the employee, keeping an eye on their health status and overall wellbeing. For others, the manager “must stay attentive [and] keep pace with the professional and personal life of their employee” (P08). This can include being alert to workload overload, as expressed by this participant: “[The manager] truly listens to our needs to determine if the pressure is too high and what she can do to accommodate us” (P10). Furthermore, to monitor employees' wellbeing in a telework context, participants revealed that their manager's strategy often involved frequent videoconference calls, either individually or in groups. Whether to discuss workload, have conversations about tasks, or provide reassurance regarding task completion. Many participants showed that their manager remained consistently present and considerate of their health status despite the distance:

If we need, say, to have a meeting with our clinical activities supervisor, at that point we will have a meeting with that person to reduce the workload, to make decisions regarding our interventions with her. […] she really tries in that regard to know where we are at, what she can do to support us, or whatever else may be needed. (P10)

Participants also expressed their appreciation for the manager's reminder of their availability for meetings, regardless of the reasons:

There are some who would be, there are employees who might feel shy about knocking on the door if it isn't explicitly stated. They might feel hesitant to do so because, deep down, they don't know how they will be received. So, the fact that the manager says, listen, my door is open, you can come anytime, you're not bothering me. (P11)

#### 6.2.3 Clarification and respect of boundaries

Clarification and respect of boundaries were significant concerns for the participants. Firstly, their comments highlight the importance, when benefiting from telework as an accommodation, of clarifying issues related to leave and vacations, particularly by ensuring a distinction between schedule flexibility of telework, sick leave, and vacation days (Category 3.1.). Indeed, working from home can create confusion between workdays and days off. There seems to be a tendency to work during times when people would typically have taken a sick day if they had to commute to the office. Thus, some participants believe that telework as an accommodation encourages individuals to gradually set aside their health and wellbeing, which could lead to burnout:

Previously, people might have been more inclined to take sick leave […] but with telework, it's almost as if you feel guilty about deciding to take a day off because you're at home, so as a result, people might end up experiencing more daily exhaustion. (P10)

On this topic, participants suggest limiting the workload to avoid the sense of obligation that telework as an accommodation can create:

Limiting, to some extent, the workload so that the person does not feel overwhelmed and indebted in their work. By taking the workload into account, so that the person does not become overly ambitious and does not lose themselves in it. By restricting the workload to certain functions in their job, it could help them feel better, I think. (P11)

However, when expectations and boundaries around telework are clearly defined, it can serve as an effective accommodation to help manage symptoms of chronic illness. Participants appreciate managers who allow them to adjust their schedules. The flexibility enables them to take necessary breaks when their symptoms are more challenging:

It is very difficult to work with a severe migraine or severe pain. So, sometimes it's about breaking the episodes; sometimes it's about being able to step away from the workplace for an hour or two. Often, that quick ability to change movement or posture, and so on, prevents longer-term absenteeism. My employer provides the flexibility to allow me to act in the moment. We are rarely alone, so there is often the possibility of having a replacement or, at least, someone to cover the need. (P03)

#### 6.2.4 Social connections

Discussions with participants also touched on issues related to social connections in telework. Trust emerged as a key factor, particularly the importance of having a trusting relationship with one's environment and manager (Category 4.1.), which enhances a worker's sense of inclusion. The distance and invisibility inherent in telework require, according to participants, mutual trust between the manager and the worker to ensure task completion and make telework a viable accommodation. For some, this trust is not just beneficial but essential, with participants stressing that trust from their workplace was a fundamental requirement:

“I wouldn't be able to work in an environment if they didn't trust me to deliver” (P01). This seems even more significant for workers with chronic illnesses. Some noted that they value being recognized for their skills rather than being defined by their health status. Therefore, it is essential for them to feel that the organization genuinely trusts their capabilities: “It may sound a bit silly […] but the first thing is that […] I was hired because I am the best person for the job. It's not because I have a work subsidy” (P06).

Secondly, privacy (Category 4.2.) emerged as a major consideration in telework, especially for workers with chronic illness or caregiving responsibilities. The importance of respecting privacy was frequently discussed by participants:

I don't think it's necessary to tell the rest of the team why, for example, we work certain hours and not others, or why we're absent for an hour for an appointment, or things like that. That's something I found has been lacking a lot in my work environment […]. I have colleagues or managers who are a bit laxer about... who can give out a lot of information for nothing, and I think that's a shame because we still need to preserve people's privacy. (P04)

Feeling free to share personal information about one's health or life situation fosters a sense of belonging: “We don't feel obligated to share, so having the freedom to choose whether or not to share personally makes me comfortable and [it makes me feel like] I belong to the company” (P05). Confidentiality regarding one's health status was crucial for participants. For example, one worker mentioned having chronic pain that prevented her from sitting at her desk for long hours while teleworking. She appreciated the possibility that her employer did not need to be aware of her condition: “There's also the fact that we don't have to tell everything to the employer. […] But that, I don't have to tell my employer, as long as the contract is delivered by the agreed time” (P02). One caregiver warns about the possible consequences of disclosure:

I had two other assignments before, well, they didn't work out, it was full-time, but I mentioned that I was a caregiver, and I think that annoyed them, knowing that sometimes I had to work 100% remotely. […] That's why you have to be cautious about what you disclose. I think even if it's 100% remote work, they don't know, and it's fine. (P09)

Thus, workers value having a manager who shows respect for everyone's privacy:

And also, we have an extraordinary manager who is very flexible, who greatly respects our private lives […] everyone agrees on that, on being flexible, we're happy, the manager is happy, it works well, and we don't question it, we respect each other's privacy. (P04)

## 7 Discussion

This qualitative study aimed to examine the experiences of individuals with chronic illness or caregiving responsibilities who utilize telework as an accommodation, with specific attention to the employee–manager relationship within this context. The results contribute to the advancement of knowledge based on two main ideas: (1) the importance of the quality of the relationship between the employee and the manager, and (2) the crucial role of trust.

### 7.1 The importance of the employee–manager relationship

Our results indicate that telework as an accommodation is subject to a negotiation process between employees and managers that varies significantly in both ease and speed (126). While some participants reported delays or the need to be persistent to secure access, others experienced favorable and rapid implementation. These initial observations align with LMX theory, which suggests that such negotiations are shaped by the quality of the dyadic relationship. Within high-quality LMX relationships, managers typically offer unwavering support and a greater willingness to assist, directly facilitating the accommodation process (75). The supportive nature of these relationships aligns with our participants' emphasis on compassionate leadership as an essential component of accommodation management. This mirrors existing research showing that a manager's empathy and openness create the safety necessary for employees to disclose their needs (40, 41, 127).

Our findings also suggest that formal accommodation policies appear secondary to the informal dynamics established between managers and employees. This points toward a gap between institutional rights and practical application. Indeed, although access to accommodation is regulated by law in many jurisdictions (128, 129), managerial discretion emerges as a determining factor in how these laws are translated into the practice. For employees with limitations, evidence shows that the granting of accommodations is influenced more by the quality of manager-employee interactions than by the objective severity of a disability (75).

However, this managerial discretion can be compromised for individuals with chronic illnesses, as workplace stereotypes and stigmatization may lead managers to question their professional competence (56, 130). Such negative perceptions can erode the relationship, reduce a manager's willingness to provide support and allow subjective factors such as perceived “likability” or “merit” to dictate access to telework (28, 31). These relational mechanisms seem to extend beyond workers with chronic illnesses to those with caregiving responsibilities. For these employees, the quality of the relationship appears to be determinant for both the availability and the efficacy of support measures (131).

Consequently, these results highlight that fostering inclusive workplaces requires organizational efforts to mitigate stigmatization and cultivate high-quality manager-employee relationships. Beyond improving access to accommodation, these relationships show potential for enhancing job satisfaction, resilience, and performance ratings (75).

### 7.2 The crucial role of trust

Our results suggest that trust is a determinant in the process of assigning and managing telework accommodations and influences the experience of individuals with chronic illness or caregiving responsibilities. Specifically, our results show that trust between an employee and their manager affects the disclosure of more sensitive information that may justify the relevance of accommodation requests as well as the employee's perceived credibility by the manager. Considering these findings in light of LMX theory, it is possible that trust, a component absent in a low-quality relationship, could explain these results.

From the manager's perspective, they might more readily question the legitimacy of the accommodation request if trust is missing in the relation with the employee. This theoretical explanatory hypothesis is consistent with findings suggesting that managers are more inclined to meet the needs of employees they trust (132). Other studies state that managers sometimes offer differential treatment in terms of accommodation measures when managers doubt the credibility of the employee (126).

On the employee's side, a lack of trust could result in withholding important details underlying the accommodation request. This lack of information sharing could negatively impact the accommodation process and hinder the support they need (27, 133). Conversely, an employee who trusts their manager is more likely to disclose the underlying details of their accommodation request, especially if they perceive support from their manager (134). This view is supported by findings showing that a perceived high-quality relationship is associated with a greater degree of disclosure to the manager among individuals suffering from inflammatory bowel disease (135). Disclosure also appears to be linked to a higher likelihood of obtaining workplace accommodations, as shown by a study involving individuals with the human immunodeficiency virus. Those who disclosed their status to their manager benefited more from accommodations compared to those who did not disclose their status (127). Building relations on trust is crucial as accommodations help retain qualified workers facing health-related challenges or caregiving responsibilities (136).

### 7.3 Practical implications and future directions

This study highlights the important role of high-quality relationships between managers and workers in effectively implementing accommodation measures. In line with LMX theory, our findings suggest that relationships characterized by trust, respect, compassion and low levels of stigma can help foster sustainable and inclusive work environments for employees managing chronic illness and caregiving responsibilities. Overall, these findings point to the importance of relational dynamics in shaping accommodation experiences and outcomes.

Although the quality of the manager–employee relation has received limited attention in the context of telework used as an accommodation, previous studies suggest that telework can create important relational challenges (137). Fewer spontaneous interactions, along with the reduced ability to interpret non-verbal cues through digital communication platforms, may make it more difficult for managers to evaluate less visible aspects of performance, including engagement, organizational commitment, and citizenship behaviors, all of which play an important role in shaping the quality of the manager–employee relationship (23). At the same time, stronger LMX relationships may help reduce the sense of professional isolation associated with telework (138). In this context, training initiatives that support managers in developing and maintaining high-quality relationships in virtual work settings could help strengthen psychological safety, improve inclusion, and reduce isolation among teleworkers receiving accommodations.

Our findings also support the idea that the practical success of telework accommodations often depends on managerial discretion, sometimes even more than on formal organizational policies (70). Our results should be viewed as a starting point for future theoretical and empirical work. Research involving larger and more diverse samples is needed to better understand how LMX quality influences access to accommodations, telework experiences, and longer-term employment outcomes. Future studies would also benefit from an intersectional approach examining how factors such as gender, disability, caregiving responsibilities, and occupational context shape accommodation experiences. Lastly, more research is needed to better understand the long-term implications of telework as a flexible and inclusive accommodation strategy and to explore how organizations can effectively support workers who rely on such arrangements.

### 7.4 Strengths and limitations

This study benefits from its rigorous qualitative design and high level of methodological transparency. The use of a bottom-up thematic analysis approach allowed themes to emerge directly from participants' lived experiences, generating grounded insights into the challenges faced by teleworkers within the employee–manager relationship. The integration of a well-established theoretical framework, LMX Theory, provided a coherent and relevant lens through which to examine the relational dimensions of workplace accommodations.

However, certain limitations should be acknowledged. Notably, the sample was predominantly composed of women, potentially introducing a gender bias. Additionally, the higher proportion of participants with chronic illness relative to caregivers may have influenced the findings. Lastly, the transferability of the results is limited by the small sample size and the specific context of the province of Quebec. Legal and institutional frameworks related to labor laws and workplace accommodations may differ across jurisdictions. These factors should therefore be considered when interpreting the findings. Nevertheless, the study provides contextualized insights that can help inform and guide future research.

## 8 Conclusion

The aim of this study was to examine the experiences of individuals with chronic illness or caregiving responsibilities who utilize telework as an accommodation, with specific attention to the employee–manager relationship within this context. Our results highlight the negotiation process involved in telework accommodations, with trust emerging as a key factor influencing both the disclosure of sensitive information and the perceived credibility of employees. They also underscore the importance of high-quality manager–employee relationships and organizational efforts to reduce workplace stigma and promote inclusion. Fostering trust and inclusive practices is essential to ensuring equitable access to telework accommodations for workers who rely on them. Further research is needed to better understand telework as a flexible and inclusive strategy to support employees with health-related challenges or caregiving responsibilities.

## Data Availability

The raw data supporting the conclusions of this article will be made available by the authors, without undue reservation.

## Footnotes

1 All verbatim quotes included in this article were originally collected in French and were translated into English for the purpose of publication. The translation aimed to preserve the meaning and nuance of participants' responses as accurately as possible.
